# Ectopic Pilonidal Disease Presenting As Anterior Leg Swelling in a Diabetic Patient: A Case Report

**DOI:** 10.7759/cureus.114215

**Published:** 2026-08-09

**Authors:** Abdulrahman Aldarkhbani, Zainab K Alkhlaif

**Affiliations:** 1 Department of General Surgery, Almoosa Health Group, Al-Ahsa, SAU

**Keywords:** case report, ectopic pilonidal disease, leg sinus, pilonidal sinus, rare location

## Abstract

Pilonidal sinus disease usually presents in the sacrococcygeal region. Its occurrence at nonsacrococcygeal sites, such as the lower limb, is exceedingly rare and may mimic other benign lesions. We present a 33-year-old man with an unusual presentation of a pilonidal sinus over his leg. Ectopic pilonidal disease should be considered in the differential diagnosis of chronic subcutaneous swellings, particularly when a punctum or sinus tract is present. Awareness of such atypical presentations ensures ectopic pilonidal disease is considered in the differential diagnosis for unusual anterior leg lesions.

## Introduction

Pilonidal disease was first described by Mayo in 1833; pilus is derived from the Latin word “hair,” and nidus means “nest” [1]. Pilonidal sinus disease (PND) represents a chronic inflammatory disorder typically localized in the region between the sacrococcygeal joint and the coccyx [2]. The term nonsacrococcygeal pilonidal sinus describes the atypical locations of pilonidal sinus other than the gluteal cleft. A systematic review published in 2023 included 447 cases of nonsacrococcygeal PND. The umbilicus accounts for 87.2% of cases, the intermammary region 3.8%, hands 3.1%, and the inguinal region 2.2%, with less frequent involvement of the face, scalp, perianal region, and neck [3]. No lower limb cases were included in the previous study. Lower limb pilonidal disease is considered rare, with few reported cases in the feet [4,5]. We present a 33-year-old man with an atypical location of a pilonidal sinus in the anterior aspect of the leg.

## Case presentation

A 33-year-old man, a known case of diabetes mellitus, presented to the surgical clinic with swelling of the right anterior leg for three weeks. The swelling was associated with mild pain and discharge. He had sought medical attention one week earlier in the emergency department, where he was treated empirically with oral amoxicillin-clavulanic acid (875/125 mg) twice daily for seven days. The patient reported improvement in his swelling, but the discharge persisted for a total of three weeks. Regarding diabetes history, he was diagnosed with type 1 diabetes mellitus in 2012, and currently maintained on a basal-bolus insulin regimen comprising once-daily basal insulin degludec (40 units) and prandial insulin aspart (15 units three times daily) before main meals. His follow-up with the ophthalmology clinic revealed mild retinopathy, while his screening results were normal with the diabetic foot clinic. No other diabetic complications were reported. The patient is a hirsute man. He denied any history of local trauma. The patient is self-employed as a coffee shop owner with prolonged daily standing.

On examination, there was anterior leg swelling, which was around 2.0 x 1.5 cm, firm, fixed, nontender, erythematous with a central punctum. There was no fluctuation or discharge at the time of examination. No systemic signs of infection were noted. Laboratory investigations, including complete blood count and serum electrolytes, were within normal limits. Due to the absence of discharge at the time of examination, no culture was taken. Glycated hemoglobin level was 7.9%. His body mass index was 32.2 kg/m^2 ^(obesity class 1).

**Figure 1:**
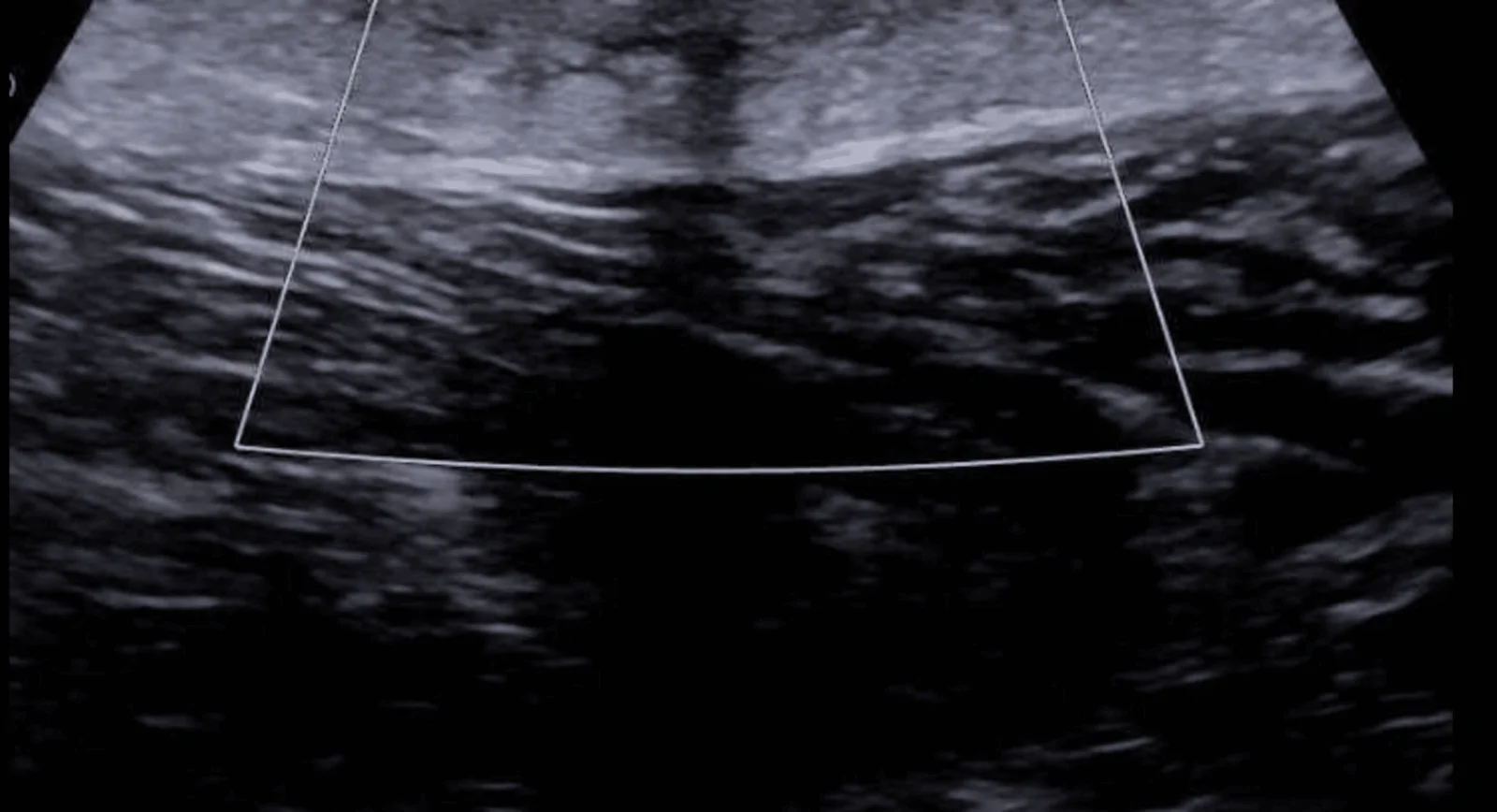
Ultrasonography image showing an area of mild subcutaneous interstitial edema

Superficial ultrasonography revealed mild subcutaneous interstitial edema without evidence of abscess or solid mass (Figure 1). With a working diagnosis of sebaceous cyst, the patient underwent complete surgical excision with primary closure. Closure was done in layers using Vicryl 3-0 (Ethicon-Johnson & Johnson, Mumbai, India), and skin was closed with Prolene 4-0 (Ethicon-Johnson & Johnson). Sutures were removed on the ninth postoperative day.

**Figure 2:**
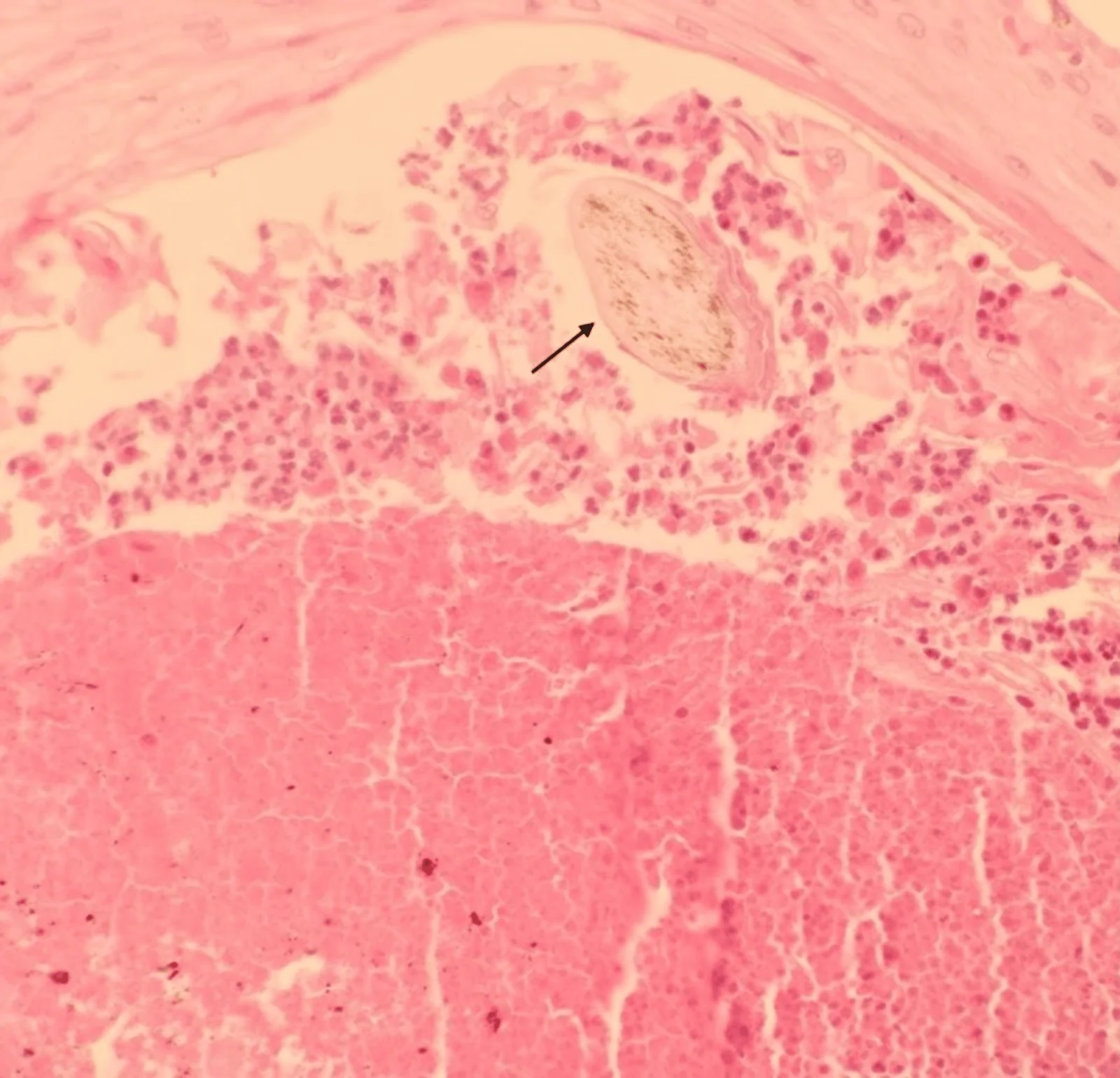
High-power image (×40 objective) of sinus tract containing hair shaft surrounded by inflammation (the arrow is pointing to the hair shaft)

**Figure 3:**
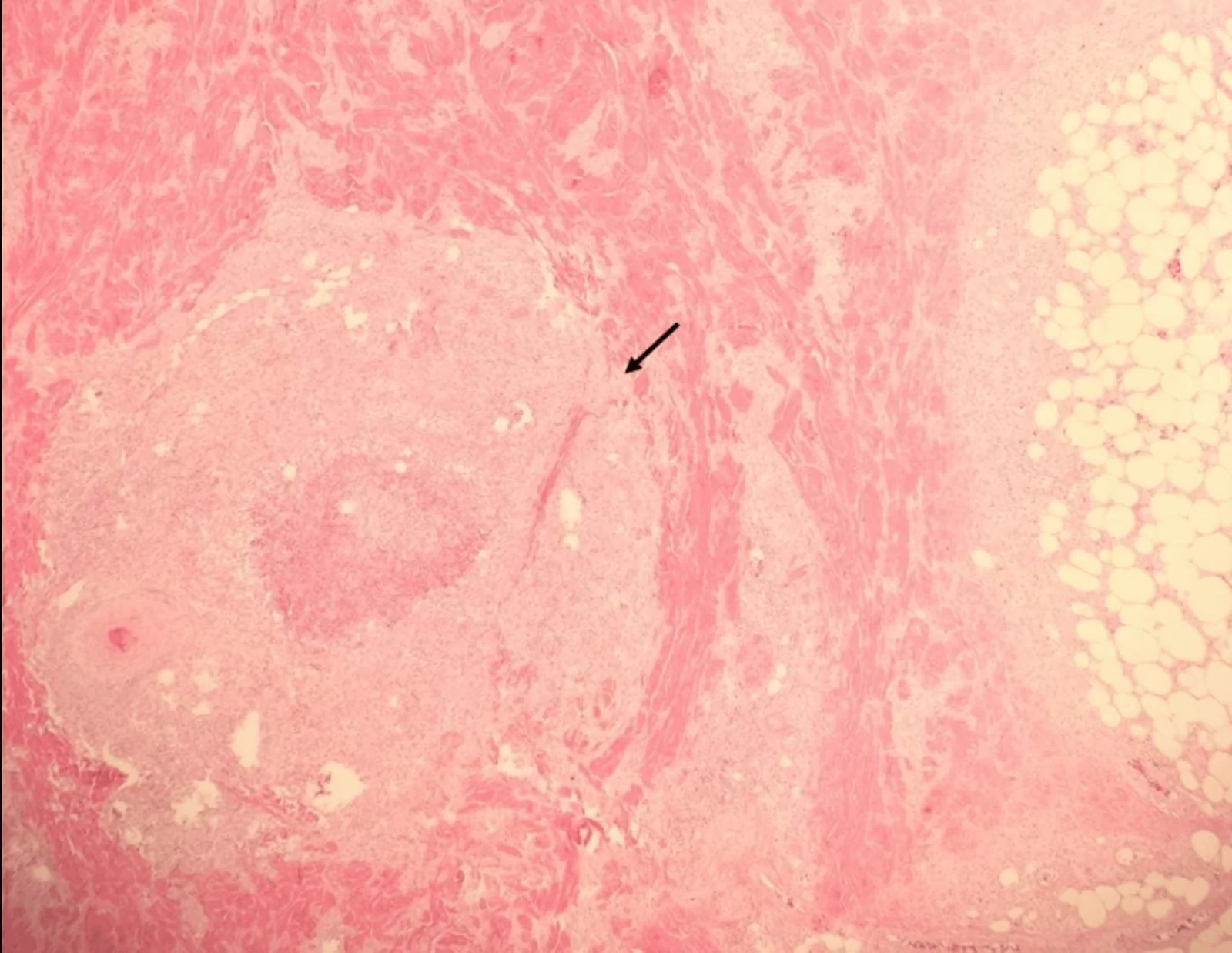
Low-power image (×10 objective) showing the area of acute inflammation and deep scarring (arrow)

Histopathology revealed hyperkeratosis of the overlying epidermis. The dermis and subcutaneous tissue contained areas of scarring and a mixed inflammatory cell infiltrate with hair shaft fragments. No multinucleated giant cells were observed. These findings confirmed the diagnosis of ectopic pilonidal disease, as shown in Figures 2, 3.

**Figure 4:**
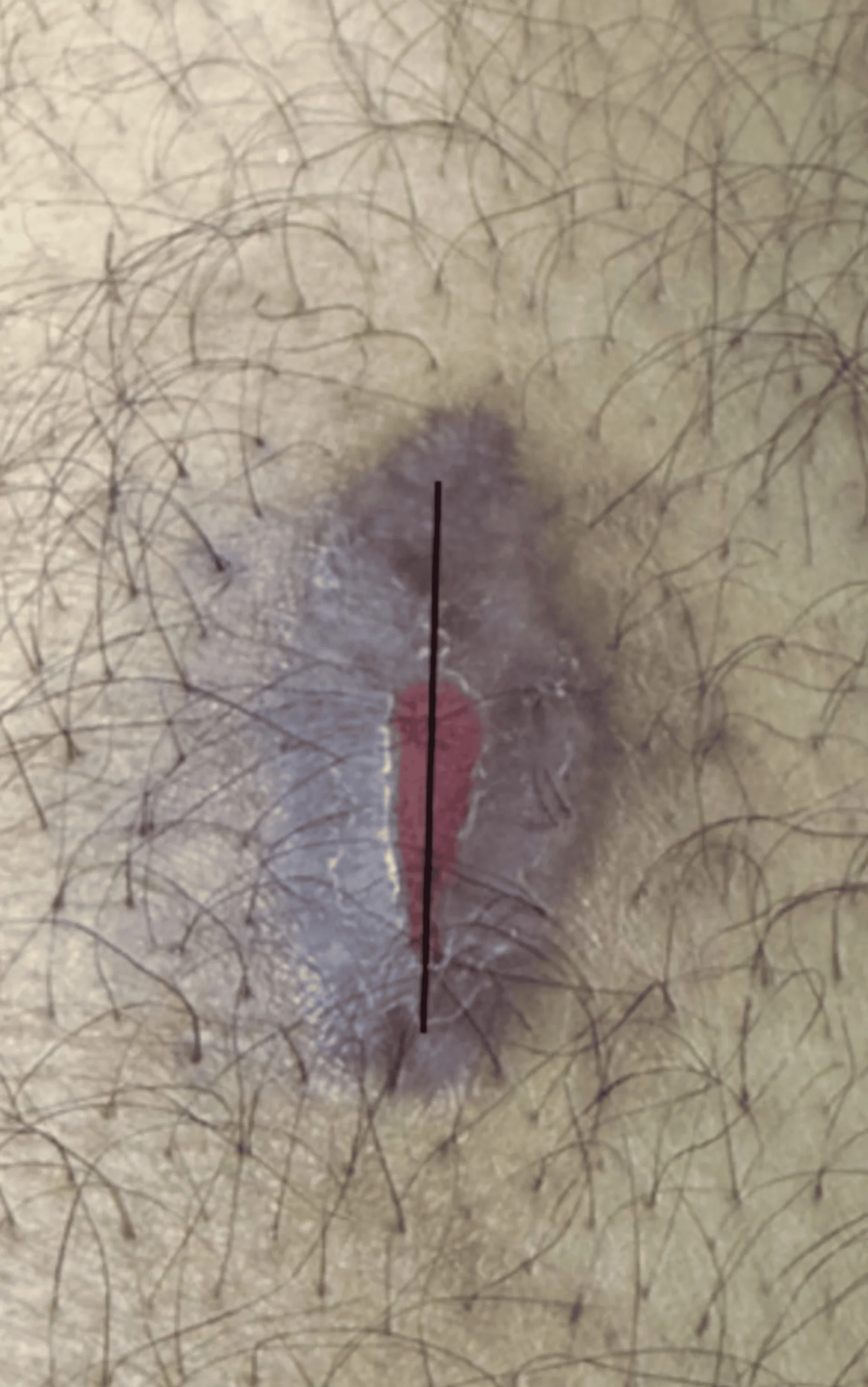
Healed leg pilonidal sinus wound at eight weeks after operation (the line represents the primary incision site)

The patient’s postoperative recovery was complicated by a surgical site infection (SSI) within the first few days following the procedure, which subsequently led to wound dehiscence. Given the disruption of the primary closure and the active infection, the wound was managed conservatively and left open to heal via secondary intention. This strategy facilitated adequate drainage, controlled the infection, and promoted the gradual formation of healthy granulation tissue. The wound healed a few weeks after surgery (Figure 4). At the one-year follow-up, no disease recurrence was observed.

## Discussion

PND affects young adults, with a higher prevalence in men. A systematic review and meta-analysis published in 2021 included 679 studies, which concluded that men are affected fourfold more than women [6]. Surprisingly, a single-institution study in Saudi Arabia showed a male-to-female ratio of 1:15 [7]. This regional disparity is possibly driven by sociocultural and methodological factors. Cultural modesty among women often leads to delayed presentation and underreporting. Additionally, community-wide screening is needed rather than a single-institution study. There are no official statistics on the exact prevalence of pilonidal disease in Saudi Arabia; however, data from a tertiary center in Al-Baha reported a prevalence of 2.9% of all surgical clinic patients [8]. Recurrence rates for PND in Saudi Arabia vary across studies, with some reporting rates as low as 6.3% after surgical management [9] and others reporting a higher recurrence rate of 22.8% in a study done in 2021 [10].

The pathogenesis of pilonidal sinus involves obstruction of the follicle opening due to folliculitis, resulting in trapped hair in the midline cleft, which triggers a foreign body reaction and granuloma [11]. Regarding our patient, the absence of giant cells and granuloma formation in the histopathology findings was attributed to the acute presentation. Anatomically, a pilonidal sinus is typically composed of two canals. The primary canal originates from the external sinus opening on the skin, usually at the midline of the gluteal cleft. It is considered the entry point for hair and debris into the subcutaneous tissues and hence the starting point of the disease. The other canal type is the secondary canal. These are epithelialized tracts that can lead to secondary openings on the skin. They form later and develop off the midline of the gluteal cleft [2,12].

​When evaluating cutaneous and subcutaneous lesions of the anterior leg, several common soft tissue entities must be distinguished from ectopic pilonidal disease. Epidermoid cysts typically present as mobile, compressible, flesh-colored subcutaneous nodules with a characteristic central punctum. Although an inflamed epidermoid cyst can present with acute swelling and thick keratinous debris mixed with pus, it almost always arises from a longstanding, preexisting nodule [13]. In contrast, our patient’s lesion lacked a chronic preexisting mass, presenting instead as a firm, noncompressible, fixed lesion of recent onset.

​A primary cutaneous abscess was also considered given the purulent discharge; however, classic abscesses develop rapidly as nonencapsulated, uniformly fluctuant liquid pus collections without a central punctum [14]. Lipomas are readily differentiated by their soft, rubbery, mobile consistency and absence of a punctum. Finally, the firm and fixed nature of the lesion warranted careful exclusion of soft-tissue malignancy, which typically exhibits rapid enlargement, ulceration, marked induration, or invasive fixed borders [15].

The use of preoperative ultrasound can help assess sinus borders and identify the presence of infection or abscess, thereby tailoring the surgical approach for each case [16]. In our case, ultrasound features were not conclusive; no sinus or abscess collection was seen. The diagnosis was made based on histopathological findings.

Sacrococcygeal PND modern management has shifted toward minimally invasive therapies, such as phenol injection, endoscopic pilonidal sinus treatment, and laser therapy, which focus on extracting foreign bodies and destroying the granulation tissue [17]. Other conventional methods are surgical excision with either primary closure or leaving the wound open for secondary healing. Primary closure with asymmetric-oblique closure techniques and full-thickness flap techniques are preferred over midline repair technique, with lower infection rates, wound dehiscence, and recurrence compared with closure with midline suture [18].

Regarding nonsacrococcygeal PND management, surgical excision and primary suturing are the mainstay of treatment, including atypical locations such as intermammary, penis, clitoris, prepuce, hand, and scalp. Conservative management in the form of removing hair tuft and daily dressing is done for umbilical PND, while perianal PND was managed by incision and leaving the wound to heal by secondary intention [3].

General risk factors for wound dehiscence are divided mainly into two categories: physician-related factors, such as level of experience, suture material, and closure technique used, and patient-related factors, such as age, wound infection, hypoalbuminemia, and emergency surgery [19]. The possible risk factors implicated in our case were relatively uncontrolled diabetes mellitus and SSI.

## Conclusions

In conclusion, PND remains a significant surgical burden that affects the young population with a higher prevalence among men. Furthermore, understanding the micropathogenesis of PND is vital for successful intervention. The disease process, driven by follicular obstruction, hair entrapment, and subsequent foreign body granuloma formation, underscores the necessity of complete debris eradication. A variety of management options are available, ranging from traditional methods such as radical excision to modern minimally invasive methods with laser or endoscopic treatment. This case emphasises the importance of considering ectopic pilonidal disease in the differential diagnosis of chronic subcutaneous swellings, even in unusual locations such as the leg. A high index of suspicion and histopathological confirmation are essential for accurate diagnosis and management.
